# Oral health status and behaviors of pregnant migrant workers in Bangkok, Thailand: a cross-sectional study

**DOI:** 10.1186/s12903-021-01732-8

**Published:** 2021-07-27

**Authors:** Wirongrong Traisuwan

**Affiliations:** Department of Dentistry, Rajavithi Hospital, 2, Phyathai Road, Bangkok, 10400 Thailand

**Keywords:** Antenatal care, Health education, Migrant workers, Oral health, Oral health behaviors, Pregnancy

## Abstract

**Background:**

There is evidence to show that immigrants have poorer oral health status than their local counterparts, and low-skilled migrant workers may also be more prone to poor oral health. This study aims to evaluate the oral health status and oral health behaviors of pregnant migrant workers compared to those of local pregnant women.

**Methods:**

A hospital-based cross-sectional study was conducted in a public general hospital in Bangkok. Pregnant migrant workers who attended the antenatal clinic were randomly enrolled at their first antenatal booking; local pregnant women were also randomly included to form a comparison group. Oral health status of all eligible pregnant women was evaluated according to the World Health Organization (WHO) protocol, and their oral health behaviors were assessed using a structured questionnaire. Oral health status and behaviors of the two pregnant groups were compared using Chi-Square test, Student’s t test, Mann–Whitney U test, Fisher’s exact test and multiple logistic regression analysis.

**Results:**

A total of 208 pregnant migrant workers and 210 local pregnant women were included. Pregnant migrant workers had significantly more dental disease than local pregnant women (DMFT mean (SD) = 5.8 (4.4) vs 4.8 (4.0), *p* = 0.014) with significant more dental decay (D mean (SD) = 5.5 (3.6) vs 3.8 (2.9), *p* < 0.001; adjusted OR 3.56 (95%CI 1.74–7.27)). Pregnant migrant workers suffered greater periodontal disease with mean (SD) CPI of 2.9 (0.6) vs 2.2 (0.5), *p* < 0.001. CPI = 3 or 4 occurred in 74.5% of migrants compared to only 22.4% of local pregnant women (adjusted OR 6.39: 95%CI 3.53–11.58). A significant greater percentage of pregnant migrants had a CPI of 4 (11.1% vs 0.5%). Pregnant migrant workers tended not to use fluoride toothpaste or dental floss and despite having 76.0% healthcare coverage, they made significantly fewer dental visits compared to local women; furthermore, the majority of them (74.5%) were under the misconception that dental treatment was prohibited during pregnancy.

**Conclusion:**

Pregnant migrant workers experienced more dental caries and periodontal disease, had less access to oral health facilities, had less knowledge of healthy oral hygiene, and had poorer oral health practices than local pregnant women. Comprehensive oral health screening and treatment during antenatal visits, together with appropriate systematic antenatal health education, could play a crucial role in improving their oral health.

## Background

The number of migrant workers in the world is increasing as a result of globalization and social and economic disruptions. Workers from less-developed economies tend to migrate to seek better jobs and higher incomes in other countries [1]. Thailand used to be a low-paced agricultural economy, but in recent decades there has been a tremendous increase in industrial and service activities with the result that the country has been transformed into a service-based and industrialized economy, and millions of workers from neighboring countries are continuing to flock into Thailand. These migrants are vulnerable groups because they typically come from less developed socioeconomic sectors and have only basic levels of education; consequently, they are compelled to perform low-skilled jobs [2]. They usually suffer long, hard working hours and do not have much time to take care of their general health. Poor oral hygiene is a major public health problem, and it could definitely be prevented merely if people acquired adequate knowledge and adopted appropriate daily hygiene practices. Several studies in various countries have demonstrated that immigrants have poorer oral health than their local counterparts [3–6]; in addition, physiologic changes during pregnancy may aggravate dental and periodontal diseases through hormonal changes and alterations in eating habits [7]. Other researches have revealed that pregnant females have poorer oral health than non-pregnant women [8–10], and pregnant women with periodontal disease may be at increased risk of preterm birth [11]. The recent Thailand national oral health survey 2017 reported 6.6 DMFT with 8.2% caries-free and CPI = 3 or 4 of 25.9% in adults at 35–44 years-old without information regarding pregnant women [12]. The present study aimed to examine the oral health status in pregnant migrant workers compared to that of their local pregnant counterparts and to study related oral health knowledge and behaviors among them.

## Methods

This was a hospital-based cross-sectional study of pregnant women who attended the antenatal clinic in Rajavithi Hospital, a general public hospital in Bangkok, Thailand, between May 2016 and August 2017. Rajavithi Hospital Ethics Committee approved the study before the commencement of enrollment. Pregnant migrant workers who attended the antenatal clinic were randomly invited to participate in the study at their first antenatal booking. Simple randomization was done via computer-generated random numbers. Inclusion criteria were pregnant migrant who: were able to verbally communicate in basic Thai; had migrated to Thailand and worked here for at least 3 months; had singleton pregnancy without underlying medical disease; and had live fetus without fetal anomaly. Local pregnant women who had their first booking in the antenatal clinic and fulfilled the relevant inclusion criteria were randomly invited to participate in the study in the comparison group. Private cases and women who were seropositive for human immunodeficiency virus (HIV), had overt diabetes or underlying medical disease were excluded. After giving informed consent, all eligible pregnant women were sent to the Dental Department, Rajavithi Hospital, for evaluation of oral health status and behaviors. A structured questionnaire was used to assess their knowledge of oral health and appropriate behaviors. The author developed the questionnaire, which was initially tested and modified in 20 pregnant migrants and 20 local pregnant women for validation. A trained research assistant reviewed medical records, retrieved demographic and clinical data, and conducted interviews using the structured questionnaire, which included queries about personal history, oral health knowledge and oral health behaviors. Migrants who demonstrated suboptimal verbal communication ability during interviewing were excluded from the study. Subsequently, a single dentist (WT) systematically examined the oral health status of every woman in accordance with the World Health Organization (WHO) protocol [13]. DMFT (decay, missing, filling, tooth) and community periodontal index (CPI) were used for tooth decay and periodontal disease evaluation respectively. CPI scores were: CPI = 0, healthy; CPI = 1, bleeding observed directly or by using a mouth mirror after probing; CPI = 2, calculus detected during probing; CPI = 3, periodontal pocket 4–5 mm.; CPI = 4, periodontal pocket 6 mm. or more; CPI = X, excluded sextant (fewer than 2 teeth presented). The greatest CPI score among all sextants was used for analysis. The dentist was blinded to the women’s ethnicity, nationality and responses to the questionnaire.

### Statistical analysis

Data was analyzed by SPSS Statistical software version 22.0. Chi square test, Fisher’s exact test and Student’s t-test were used for relevant variables while Mann–Whitney U test was used if the dataset was not normally distributed. Multiple logistic regression analysis was used to adjust potential confounding variables.

## Results

A total of 220 pregnant women were enrolled in each group. In the pregnant migrant worker group, 10 women with suboptimal verbal communication and 2 women with underlying diabetes were excluded. In the local pregnant women group, 7 women who were private cases and 3 who had underlying diabetes were also excluded. Eventually, 208 pregnant migrant workers and 210 local pregnant women were included in the analysis.

In the pregnant migrant workers group, 74.0% had migrated from Myanmar, 14.9% from the People Republic of Lao, 6.3% from Cambodia and 4.8% from other countries. The mean (SD) time of residence in Thailand was 6.9 (3.8) years. The largest proportion of the migrants worked as household assistants (38.0%), followed by shop assistants (23.1%), contractors (17.3%), construction workers (12.0%) and factory workers (8.2%). Compared to local pregnant women, migrant workers had significantly lower monthly household incomes. In the migrant group, 76.0% had healthcare cost coverage from the national migrant workers’ health welfare program (69.7%) or individual employer healthcare support (6.3%), while all of the local pregnant women had healthcare cost coverage under the national universal social healthcare or social security program. All healthcare programs include basic dental examination and treatment cost coverage; thus, the majority of eligible women did not have to pay for a dental visit. Interviewing and evaluation of oral health occurred in 27.9%, 32.2% and 39.9% of participants in the first, second and third trimester respectively in the migrant worker group, and in 51.6%, 35.7% and 12.7% respectively in the local pregnant group. Demographic characteristics of the two groups are presented in Table 1. There are significant differences in age, monthly household income and gestational age at participation between the two groups. Therefore, these variables were included in multiple logistic regression analysis to adjust the odds ratios (ORs) of oral health status.

**Table 1:** Demographic characteristic of pregnant migrant workers and local pregnant women

	Pregnant migrant workers n = 208 (%)	Local pregnant women n = 210 (%)	*p* value
Age (years) mean ± SD	27.5 ± 4.9	26.2 ± 6.1	0.017*^T^
BMI (kg/m^2^) mean ± SD	22.2 ± 2.8	21.7 ± 4.0	0.188^ T^
Smoking N (%)	0 (0)	2 (1.0)	
Previous preterm birth N (%)	3 (1.4)	5 (2.4)	0.724^F^
Work status N (%)			
Housewife	0 (0)	74 (35.4)	
Company employee	0 (0)	26 (12.4)	
Government employee	0 (0)	13 (6.2)	
Private business	0 (0)	13 (6.2)	
Household assistant	79 (38.0)	0 (0)	
Shop assistant	48 (23.1)	19 (9.0)	
Contractor	36 (17.3)	54 (25.7)	
Construction worker	25 (12.0)	3 (1.4)	
Factory worker	17 (8.2)	8 (3.8)	
Others	3 (1.4)	0 (0)	
Monthly household income (Baht) Median (min–max)	13,750 (5000–27,000)	20,000 (5000–81,600)	< .001*^M^
Gestational age at participation N (%)			< .001*^C^
1st trimester	58 (27.9)	110 (51.6)	
2nd trimester	67 (32.2)	76 (35.7)	
3rd trimester	83 (39.9)	27 (12.7)	

C = *p* value from Chi-Square test, F = *p* value from Fisher’s exact Test, M = *p* value from Mann–Whitney U Test and T = *p* value form Student’s t-test

*Significant at *p* value < 0.05

Oral health examination revealed that overall pregnant women had considerable dental disease with 5.3 DMFT (included caries-free women). Pregnant migrant workers were less likely to be caries-free (9.1% vs 14.8%) and had higher DMFT scores than local pregnant women (mean (SD) DMFT = 5.8 (4.4) vs 4.8 (4.0), *p* = 0.014) (Table 2). In pregnant women who had caries, migrant workers had significant more tooth decay (D mean (SD) = 5.5 (3.6) vs 3.8 (2.9), *p* < 0.001; adjusted OR 3.56 (95%CI 1.74–7.27)) (Tables 2, 3). Gingival inflammation was observed among all of the pregnant women, with all having a CPI of at least 1 (overall mean (SD) CPI of 2.5 (0.6)); although higher CPI scores were observed among migrant women (2.9 (0.6) vs 2.2 (0.5), *p* < 0.001). A higher proportion of migrant women had a CPI of 3 or 4 (74.5%) compared to the local women (22.4%) (adjusted OR 6.39: 95%CI 3.53–11.58). In addition, 11.1% of the pregnant migrant workers had CPI = 4 compared with only 0.5% of local pregnant women (Tables 4, 5). After statistical adjustment for age, household income, and gestational age at participation, pregnant migrant workers still had significantly greater dental and periodontal diseases compared to local pregnant women (Tables 3, 5).

**Table 2:** Dental disease in pregnant migrant workers and local pregnant women

Dental disease	Total (n = 418)		Pregnant women				*p* value
			Migrant (n = 208)		Local (n = 210)		
	n	%	n	%	n	%	
Caries-free	50	12.0%	19	9.1%	31	14.8%	0.076^C^
Tooth decay	333	79.7%	185	88.9%	148	70.5%	< 0.001*^C^
Tooth missing	103	24.6%	45	21.6%	58	27.5%	0.156^C^
Tooth filling	145	34.7%	42	20.2%	103	49.0%	< 0.001*^C^
D (mean ± SD)	4.7 ± 3.4		5.5 ± 3.6		3.8 ± 2.9		< 0.001*^M^
M (mean ± SD)	1.8 ± 1.3		1.5 ± 1.0		2.0 ± 1.5		0.041*^M^
F (mean ± SD)	3.2 ± 2.5		3.2 ± 2.5		3.1 ± 2.5		0.386^ M^
DMFT (mean ± SD)	5.3 ± 4.2		5.8 ± 4.4		4.8 ± 4.0		0.014*^M^

C = *p* value from Chi-Square test, F = *p* value from Fisher’s exact Test, M = *p* value from Mann–Whitney U Test and T = *p* value form Student’s t-test

*Significant at *p* value < 0.05

**Table 3:** Multiple logistic regression analysis of dental disease in pregnant migrants and local pregnant women

	Crude OR	(95% CI)	*p* value	Adjusted OR**	(95% CI)	*p* value
Tooth decay (D)						
Migrant	3.37	(1.99–5.70)	< 0.001*	3.56	(1.74–7.27)	< 0.001*
Local	1			1		
Tooth missing (M)						
Migrant	0.72	(0.46–1.13)	0.157	0.67	(0.36–1.23)	0.194
Local	1			1		
Tooth filling (F)						
Migrant	0.26	(0.17–0.40)	< 0.001*	0.32	(0.18–0.58)	< 0.001*
Local	1			1		

OR = Odds Ratio

**Adjusted by age, monthly household income and gestational age at participation

*Significant at *p* value < 0.05

**Table 4:** Periodontal disease in pregnant migrant workers and local pregnant women assessed with community periodontal index (CPI)

Periodontal disease	Total (n = 418)		Pregnant women				*p* value
			Migrant (n = 208)		Local (n = 210)		
	n	%	n	%	n	%	
CPI = 0	0	0%	0	0%	0	0%	
CPI = 1	9	2.2%	0	0%	9	4.3%	0.004*^F^
CPI = 2	207	49.5%	53	25.5%	154	73.3%	< 0.001*^C^
CPI = 3	178	42.6%	132	63.5%	46	21.9%	< 0.001*^C^
CPI = 4	24	5.7%	23	11.1%	1	0.5%	< 0.001*^C^
CPI = 3 or 4	202	48.3%	155	74.5%	47	22.4%	< 0.001*^C^
CPI Mean ± SD	2.5 ± 0.6		2.9 ± 0.6		2.2 ± 0.5		< 0.001*^M^

C = *p* value from Chi-Square test, F = *p* value from Fisher’s exact Test and M = *p* value from Mann–Whitney U Test

*Significant at *p* value < 0.05

**Table 5:** Multiple logistic regression analysis of periodontal disease in pregnant migrants and local pregnant women

	Crude OR	(95% CI)	*p* value	Adjusted OR**	(95% CI)	*p* value
CPI = 2						
Migrant	0.12	(0.08–0.19)	< 0.001*	0.20	(0.11–0.35)	< 0.001*
Local	1			1		
CPI = 3						
Migrant	6.19	(4.02–9.54)	< 0.001*	3.64	(2.05–6.47)	< 0.001*
Local	1			1		
CPI = 4						
Migrant	25.98	(3.47–194.29)	0.002*	17.80	(2.05–154.34)	0.009*
Local	1			1		
CPI = 3 or 4						
Migrant	10.14	(6.47–15.91)	< 0.001*	6.39	(3.53–11.58)	< 0.001*
Local	1			1		

OR = odds ratio

**Adjusted by age, monthly household income and gestational age at participation

*Significant at *p* value < 0.05

Oral health behaviors are shown in Table 6. Nearly all pregnant women brushed their teeth with toothpaste twice daily or more frequently; however, 76.4% of the migrants had never heard about fluoride-fortified toothpaste, 77.9% did not recognize the protective effect of fluoride for dental decay prevention, only 18.3% used fluoride toothpaste regularly, and 80.3% did not know whether their daily toothpaste was fortified with fluoride or not. Only 2 women (1.0%) in the migrant group and 9 (4.3%) in the local pregnant group used dental floss daily. Of all the included pregnant women (both migrants and locals), 51.7% used toothpicks, 13.6% consumed daily sweet snacks, and 8.9% consumed soda drinks on a daily basis.

**Table 6:** Oral health behaviors and oral health knowledge of pregnant migrant workers and local pregnant women

Oral health behaviors and knowledge n (%)	Pregnant migrants (n = 208)	Local pregnant women (n = 210)	*p* value
Brushed teeth twice daily or more often	201 (96.6)	206 (98.1)	0.351
Did not know about fluoride toothpaste	159 (76.4)	20 (9.5)	< 0.001*
Was ignorant regarding the protective effect of fluoride toothpaste	46 (22.1)	188 (89.5)	< 0.001*
Used fluoride toothpaste regularly	38 (18.3)	167 (79.5)	< 0.001*
Did not know whether the toothpaste they used on a daily basis was fluorinated	167 (80.3)	38 (18.1)	< 0.001*
Did not appreciate the need to use fluoride toothpaste	172 (82.7)	33 (15.7)	< 0.001*
Did not know about dental floss	115 (55.3)	34 (16.2)	< 0.001*
Knew about dental floss but had never used it	64 (30.8)	118 (56.2)	< 0.001*
Had used dental floss	26 (12.5)	43 (20.5)	0.028*
Used dental floss daily	2 (1.0)	9 (4.3)	0.344^F^
Used a toothpick	96 (46.2)	120 (57.1)	0.025*
Consumed sweets on a daily basis	28 (13.5)	29 (13.8)	0.917
Consumed soda drinks on a daily basis	20 (9.6)	17 (8.1)	0.584
Had never visited a dentist or had done so less than once in a year	127 (61.1)	22 (10.5)	< 0.001*
Was aware of increased risk of tooth decay and periodontal disease during pregnancy	55 (26.4)	135 (64.3)	< 0.001*
Believed that pregnant women should not get oral treatment	155 (74.5)	92 (43.8)	< 0.001*

*p* value from Chi-Square test and F = *p* value from Fisher’s exact Test

*Significant at *p* value < 0.05

Migrants made significantly fewer dental visits compared to local women, with 61.1% of migrants never having made a visit or having visited less frequently than once a year. Regarding knowledge, 73.6% migrants were not aware that pregnancy aggravated dental decay and periodontal disease, and 74.5% believed that dental treatment could not be done during pregnancy.

## Discussion

Our results showed that pregnant migrant workers had significantly more dental and periodontal diseases compared to local pregnant women. Migrants had significantly more dental caries (88.9 vs 70.5%) with greater DMFT (5.8 vs 4.8). The World Health Organization (WHO) has proposed a global goal for dental caries at 12 years of age of no more than 3 DMFT [14]; however, both pregnant groups suffered more dental caries than the DMFT target figure, and our data highlights dental caries as a persistent major health problem in pregnancy. As for periodontal disease, 74.5% of pregnant migrants had a CPI of 3 or 4 compared to only 22.4% in local group, and 11.1% suffered severe disease with deep pockets (CPI = 4). Compare with the results of 2017 Thailand national oral health survey among Thai adults at 35–44 years-old [12], local pregnant women seem to have higher proportion of person with caries-free (14.8% vs 8.2%) with lesser DMFT score (4.8 vs 6.6), but the proportions of person with a CPI of 3 or 4 are similar (22.4% vs 25.9%). However, the figures cannot be directly compared because of the discrepancy in age groups, pregnancy status and the heterogeneity of individual characteristics (gender, geographic, underlying disease etc.) that inherited among this study and the national survey. Several studies have revealed more dental caries and periodontal disease in pregnant women than in non-pregnant women [8–10] and the oral health problem is even more obvious in pregnant women who are of low socio-economic status [15, 16]. The present study showed that being a migrant worker is another risk factor for poor oral health in pregnant women.

The prevalence of dental caries and periodontal disease in pregnant women may vary among different populations. Deghatipour et al. reported mean (SD) DMFT of 10.34 (5.10) in pregnant women in Varamin, Iran [16], and this is substantially higher than in our study. In contrast, a hospital-based study in Sudan reported mean DMFT of 3.49 in pregnant women who were > 20 years old [17]. Payal et al. [9] reported mean CPI score of 2.16 in pregnant women in central India, which is similar to our results; however, our study revealed that 74.5% of pregnant migrant workers had CPI of 3 or 4, which is much greater than the 36% reported from the study of Ethiopian immigrants [5]. Ethnics, food culture, eating habits, socioeconomic status, and healthcare systems may contribute to the differences in the disease prevalence.

Pregnant migrant workers have a severe lack of appropriate knowledge and practices for good oral hygiene. Although pregnant migrants brush their teeth once, twice or more often daily, which is compatible with the ADA (American Dental Association) recommendation [18], the majority of them are unaware of the advantages of fluoride in preventing dental decay; consequently, they do not realize that they should routinely use fluoride toothpaste. The ADA also recommends flossing teeth at least once daily, but neither pregnant group used dental floss on a regular basis. We emphasize the importance of systematically using fluoride toothpaste and dental floss in antenatal health education, as inadequate knowledge and poor practices may aggravate dental caries and periodontal disease. About 1 in 10 of both pregnant groups still consumed sweets and sodas daily, and this certainly increases the risk of dental and periodontal problems. Intake of sweets and soda intake also increases the chance of maternal overweight and gestational diabetes [19]; thus, these eating habits should be discouraged through intensive health education and behavioral modification.

In addition, migrant workers were afraid that dental treatment could harm their fetus and were under the misconception that it is prohibited during pregnancy; consequently, they may not seek dental treatment during pregnancy, and this could lead to rapid disease progression. Migrants also made significantly fewer dental visits than local pregnant women. Despite having health welfare coverage, time and economic constraints in terms of leaving work for a dental visit may have a major detrimental impact on the migrant workers’ access to dental treatment. Erdsiek et al. [20] have shown that, in Germany, migrant status is associated with a reduced chance of attending dental check-ups, independent of demographic, socioeconomic factors and type of health insurance.

Antenatal health care provides opportunities to supply vulnerable pregnant migrants with pertinent information about appropriate hygiene practices, and ways in which they could alter their attitude to daily oral health habits. In addition, oral health evaluation and relevant treatments can also be accomplished during antenatal visits. A comprehensive antenatal oral health protocol that includes relevant health education and inherent dental services (both screening and treatment) is crucial to improving oral health in pregnant women [21], especially those who are part of a vulnerable population. Many groups have developed specific oral health education [22, 23] and screening programs [22, 24] for pregnant women during antenatal periods that have yielded favorable results.

This study had a major limitation in that we included only pregnant migrants who could communicate orally in Thai. Migrants who are unable to communicate in a local language may suffer more barriers in accessing oral health facilities and receiving appropriate oral health information, and they may subsequently have more oral health problems. In addition, the difference in gestational age at participation may affect the CPI results despite multiple logistic regression model adjustment. Lastly, the study was conducted in a single hospital; therefore, the results are not representing the whole population in Bangkok.

## Conclusion

Pregnant migrant workers experienced more dental caries and periodontal disease, had less access to oral health facilities, had less knowledge of healthy oral hygiene, and had poorer oral health practices than local pregnant women. Comprehensive oral health screening and treatment during antenatal visits, together with appropriate systematic health education, could play a crucial role in improving their oral health during pregnancy and beyond.

## Data Availability

The datasets used during the current study are available from the corresponding author on reasonable request.

## Abbreviations

ADA: American Dental Association
CPI: Community periodontal index
DMFT: Decay, missing, filling, tooth
WHO: World Health Organization
